# Zwitterionic 4-carb­oxy-2-(1-methyl­pyridin-1-ium-4-yl)-1*H*-imidazole-5-carboxyl­ate

**DOI:** 10.1107/S1600536811055644

**Published:** 2012-01-11

**Authors:** Dao-Sen Liu, Jun-Guo Fang

**Affiliations:** a Communication and Electronic Engineering Institute, Qiqihar University, 161006 Qiqihar, Heilongjiang, People’s Republic of China; b The First High School of Laha, 161342, Nehe, Heilongjiang, People’s Republic of China

## Abstract

In the title zwitterionic mol­ecule, C_11_H_9_N_3_O_4_, the imidazole and pyridine rings form a dihedral angle of 2.60 (2)°. An intra­molecular O—H⋯O hydrogen bond occurs. In the crystal, pairs of N—H⋯O hydrogen bonds link the mol­ecules into inversion dimers. Weak inter­molecular C—H⋯O inter­actions further consolidate the crystal packing.

## Related literature

For the use and related structures of the multifunctional connector 2-(pyridin-4-yl)-1*H*-imidazole-4,5-dicarb­oxy­lic acid in coordination chemistry, see: Sun *et al.* (2006 ▶); Li *et al.* (2009**a* ▶,b* ▶); Jing *et al.* (2010**a* ▶,b* ▶); Zhou *et al.* (2011 ▶). For the synthesis of the title compound, see: Lebedev *et al.* (2007 ▶).
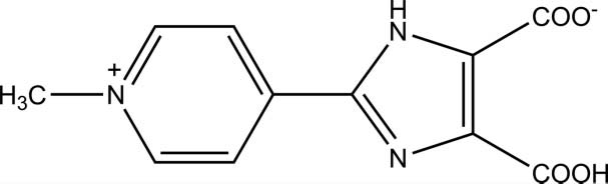


## Experimental

### 

#### Crystal data

C_11_H_9_N_3_O_4_
*M* *_r_* = 247.21
Monoclinic, 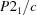

*a* = 5.4407 (17) Å
*b* = 8.634 (3) Å
*c* = 22.317 (7) Å
β = 96.327 (4)°
*V* = 1042.0 (6) Å^3^
*Z* = 4
Mo *K*α radiation
μ = 0.12 mm^−1^
*T* = 298 K
0.20 × 0.15 × 0.12 mm

#### Data collection

Bruker SMART APEXII CCD area-detector diffractometer
Absorption correction: multi-scan (*SADABS*; Bruker, 2005 ▶) *T*_min_ = 0.976, *T*_max_ = 0.985
4932 measured reflections
1816 independent reflections
1315 reflections with *I* > 2σ(*I*)
*R*_int_ = 0.039

#### Refinement

*R*[*F* ^2^ > 2σ(*F* ^2^)] = 0.057
*wR*(*F* ^2^) = 0.167
*S* = 1.10
1816 reflections
165 parameters
H-atom parameters constrained
Δρ_max_ = 0.23 e Å^−3^
Δρ_min_ = −0.26 e Å^−3^

### 

Data collection: *APEX2* (Bruker, 2005 ▶); cell refinement: *SAINT* (Bruker, 2005 ▶); data reduction: *SAINT*; program(s) used to solve structure: *SHELXTL* (Sheldrick, 2008 ▶); program(s) used to refine structure: *SHELXTL*; molecular graphics: *SHELXTL*; software used to prepare material for publication: *SHELXTL*.

## Supplementary Material

Crystal structure: contains datablock(s) global, I. DOI: 10.1107/S1600536811055644/cv5222sup1.cif

Structure factors: contains datablock(s) I. DOI: 10.1107/S1600536811055644/cv5222Isup2.hkl

Supplementary material file. DOI: 10.1107/S1600536811055644/cv5222Isup3.cml

Additional supplementary materials:  crystallographic information; 3D view; checkCIF report

## Figures and Tables

**Table 1:** Hydrogen-bond geometry (Å, °)

*D*—H⋯*A*	*D*—H	H⋯*A*	*D*⋯*A*	*D*—H⋯*A*
N1—H1⋯O1^i^	0.86	1.91	2.764 (3)	174
O3—H3⋯O2	0.82	1.64	2.461 (3)	176
C7—H7⋯O1^i^	0.93	2.24	3.145 (4)	165
C8—H8⋯O3^ii^	0.93	2.58	3.363 (4)	142
C10—H10⋯O4^iii^	0.93	2.32	3.172 (4)	152
C11—H11*C*⋯O4^iii^	0.96	2.35	3.223 (4)	150

Symmetry codes: (i) 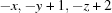; (ii) 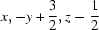; (iii) 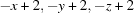.

**Fig. 1.**
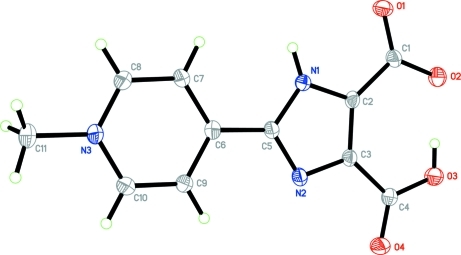
The molecular structure of (I) with displacement ellipsoids drawn at the 30% probability level.

**Fig. 2.**
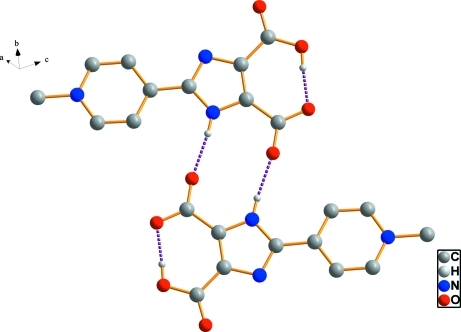
The hydrogen-bonded (dashed lines) dimer in (I). H atoms not included in hydrogen bonding have been omitted for clarity.

## supplementary crystallographic information

### Comment

The construction of metal complexes based on 2-(pyridin-4-yl)-1*H*-imidazole-4,5-dicarboxylic acid has attracted much attention due to their intriguing topologies and potential applications in many fields (Sun *et al.*, 2006; Li *et al.*, 2009**a*,b*; Jing *et al.*, 2010**a*,b*; Zhou *et al.*, 2011). In the search for new 2-(pyridin-4-yl)-1*H*-imidazole-4,5-dicarboxylic acids, the title compound, (I), has been synthesized. Herewith we report its crystal structure.

In (I) (Fig. 1), the C1-containing carboxyl group is deprotonated and forms an intramolecular hydrogen bond with the neighboring C4-containing carboxyl group. The dihedral angle formed by the imidazole and pyridyl rings is 2.60 (2) °. In the crystal structure, intermolecular N—H···O hydrogen bonds (Table 1) link the molecules into centrosymmetric dimers (Fig. 2). Weak intermolecular C—H···O interactions (Table 1) consolidate further the crystal packing.

### Experimental

The title compound was synthesized according to the method reported in the literature (Lebedev *et al.*, 2007). Light yellow single crystals suitable for X-ray diffraction were obtained by slow evaporation of an acetonitrile solution of the compound.

### Refinement

All H atoms were placed in calculated positions (C—H = 0.93—0.96 Å, N—H = 0.86 Å and O—H = 0.82 Å) and refined as riding. For those bound to C_aryl_ and N, *U*_iso_(H) = 1.2 *U*_eq_(C, N), while for those bound to C_methyl_ and O, *U*_iso_(H) = 1.5 *U*_eq_(C, O).

### Crystal data

**Table:**

C_11_H_9_N_3_O_4_	*F*(000) = 512
*M**_r_* = 247.21	*D*_x_ = 1.576 Mg m^−^^3^
Monoclinic, *P*2_1_/*c*	Mo *K*α radiation, λ = 0.71073 Å
Hall symbol: -P 2ybc	Cell parameters from 1531 reflections
*a* = 5.4407 (17) Å	θ = 2.5–26.9°
*b* = 8.634 (3) Å	µ = 0.12 mm^−^^1^
*c* = 22.317 (7) Å	*T* = 298 K
β = 96.327 (4)°	Block, light yellow
*V* = 1042.0 (6) Å^3^	0.20 × 0.15 × 0.12 mm
*Z* = 4	

### Data collection

**Table:**

Bruker SMART APEXII CCD area-detector diffractometer	1816 independent reflections
Radiation source: fine-focus sealed tube	1315 reflections with *I* > 2σ(*I*)
graphite	*R*_int_ = 0.039
φ and ω scans	θ_max_ = 25.0°, θ_min_ = 1.8°
Absorption correction: multi-scan (*SADABS*; Bruker, 2005)	*h* = −6→6
*T*_min_ = 0.976, *T*_max_ = 0.985	*k* = −10→9
4932 measured reflections	*l* = −26→24

### Refinement

**Table:**

Refinement on *F*^2^	Primary atom site location: structure-invariant direct methods
Least-squares matrix: full	Secondary atom site location: difference Fourier map
*R*[*F*^2^ > 2σ(*F*^2^)] = 0.057	Hydrogen site location: inferred from neighbouring sites
*wR*(*F*^2^) = 0.167	H-atom parameters constrained
*S* = 1.10	*w* = 1/[σ^2^(*F*_o_^2^) + (0.0889*P*)^2^ + 0.001*P*] where *P* = (*F*_o_^2^ + 2*F*_c_^2^)/3
1816 reflections	(Δ/σ)_max_ = 0.001
165 parameters	Δρ_max_ = 0.23 e Å^−^^3^
0 restraints	Δρ_min_ = −0.26 e Å^−^^3^

### Special details

**Table:**

Geometry. All e.s.d.'s (except the e.s.d. in the dihedral angle between two l.s. planes) are estimated using the full covariance matrix. The cell e.s.d.'s are taken into account individually in the estimation of e.s.d.'s in distances, angles and torsion angles; correlations between e.s.d.'s in cell parameters are only used when they are defined by crystal symmetry. An approximate (isotropic) treatment of cell e.s.d.'s is used for estimating e.s.d.'s involving l.s. planes.
Refinement. Refinement of *F*^2^ against ALL reflections. The weighted *R*-factor *wR* and goodness of fit *S* are based on *F*^2^, conventional *R*-factors *R* are based on *F*, with *F* set to zero for negative *F*^2^. The threshold expression of *F*^2^ > σ(*F*^2^) is used only for calculating *R*-factors(gt) *etc*. and is not relevant to the choice of reflections for refinement. *R*-factors based on *F*^2^ are statistically about twice as large as those based on *F*, and *R*- factors based on ALL data will be even larger.

### Fractional atomic coordinates and isotropic or equivalent isotropic displacement parameters (Å^2^)

**Table:**

	*x*	*y*	*z*	*U*_iso_*/*U*_eq_	
C1	0.0755 (6)	0.6050 (4)	1.10251 (13)	0.0317 (8)	
C2	0.2747 (5)	0.6905 (3)	1.07599 (12)	0.0267 (7)	
C3	0.4543 (5)	0.7976 (3)	1.09778 (12)	0.0274 (7)	
C4	0.4977 (6)	0.8681 (3)	1.15866 (13)	0.0322 (8)	
C5	0.5063 (5)	0.7576 (3)	1.00524 (12)	0.0273 (7)	
C6	0.6061 (5)	0.7649 (3)	0.94726 (12)	0.0280 (7)	
C7	0.5034 (6)	0.6830 (3)	0.89683 (13)	0.0317 (8)	
H7	0.3659	0.6203	0.8992	0.038*	
C8	0.6059 (6)	0.6954 (4)	0.84385 (13)	0.0342 (8)	
H8	0.5370	0.6407	0.8102	0.041*	
C9	0.8120 (6)	0.8566 (3)	0.94116 (13)	0.0318 (8)	
H9	0.8844	0.9126	0.9741	0.038*	
C10	0.9089 (6)	0.8656 (4)	0.88748 (13)	0.0341 (8)	
H10	1.0469	0.9271	0.8840	0.041*	
C11	0.9080 (7)	0.7973 (5)	0.78104 (14)	0.0559 (11)	
H11A	0.8038	0.8626	0.7543	0.084*	
H11B	0.9161	0.6960	0.7636	0.084*	
H11C	1.0711	0.8409	0.7873	0.084*	
N1	0.3130 (4)	0.6680 (3)	1.01751 (10)	0.0280 (6)	
H1	0.2289	0.6073	0.9925	0.034*	
N2	0.5960 (5)	0.8392 (3)	1.05351 (10)	0.0299 (6)	
N3	0.8048 (5)	0.7854 (3)	0.83951 (10)	0.0322 (7)	
O1	−0.0546 (4)	0.5157 (3)	1.06919 (9)	0.0433 (7)	
O2	0.0558 (5)	0.6265 (3)	1.15773 (9)	0.0483 (7)	
O3	0.3582 (5)	0.8213 (3)	1.19912 (9)	0.0486 (7)	
H3	0.2630	0.7543	1.1845	0.073*	
O4	0.6553 (4)	0.9671 (3)	1.17050 (9)	0.0454 (7)	

### Atomic displacement parameters (Å^2^)

**Table:**

	*U*^11^	*U*^22^	*U*^33^	*U*^12^	*U*^13^	*U*^23^
C1	0.0347 (19)	0.0341 (18)	0.0278 (16)	−0.0051 (15)	0.0098 (13)	0.0006 (14)
C2	0.0324 (18)	0.0282 (17)	0.0203 (15)	0.0011 (13)	0.0072 (12)	−0.0009 (12)
C3	0.0334 (18)	0.0273 (16)	0.0222 (15)	−0.0023 (13)	0.0057 (13)	−0.0002 (12)
C4	0.0350 (19)	0.0365 (19)	0.0255 (16)	−0.0037 (15)	0.0053 (14)	−0.0007 (14)
C5	0.0307 (17)	0.0282 (16)	0.0238 (15)	−0.0023 (13)	0.0058 (12)	0.0019 (12)
C6	0.0310 (17)	0.0283 (16)	0.0253 (16)	0.0017 (14)	0.0046 (13)	0.0022 (13)
C7	0.0342 (18)	0.0344 (18)	0.0275 (16)	−0.0071 (14)	0.0082 (13)	0.0003 (13)
C8	0.0351 (19)	0.0407 (19)	0.0269 (16)	−0.0054 (15)	0.0042 (14)	−0.0034 (14)
C9	0.0350 (19)	0.0355 (18)	0.0260 (16)	−0.0074 (14)	0.0076 (13)	−0.0052 (13)
C10	0.0321 (19)	0.0382 (19)	0.0324 (17)	−0.0081 (14)	0.0058 (14)	−0.0021 (14)
C11	0.061 (3)	0.080 (3)	0.0312 (19)	−0.022 (2)	0.0210 (17)	−0.0111 (18)
N1	0.0318 (15)	0.0298 (14)	0.0232 (13)	−0.0060 (11)	0.0059 (11)	−0.0024 (10)
N2	0.0347 (15)	0.0308 (14)	0.0250 (13)	−0.0035 (11)	0.0075 (11)	0.0020 (11)
N3	0.0327 (16)	0.0404 (16)	0.0248 (13)	−0.0042 (12)	0.0088 (11)	−0.0014 (11)
O1	0.0483 (15)	0.0477 (14)	0.0358 (13)	−0.0221 (11)	0.0127 (11)	−0.0106 (11)
O2	0.0573 (16)	0.0652 (17)	0.0256 (12)	−0.0240 (13)	0.0190 (10)	−0.0065 (11)
O3	0.0550 (16)	0.0661 (17)	0.0265 (12)	−0.0264 (13)	0.0135 (11)	−0.0101 (11)
O4	0.0523 (16)	0.0483 (14)	0.0358 (13)	−0.0199 (12)	0.0062 (11)	−0.0091 (11)

### Geometric parameters (Å, °)

**Table:**

C1—O1	1.238 (3)	C7—C8	1.366 (4)
C1—O2	1.262 (4)	C7—H7	0.9300
C1—C2	1.487 (4)	C8—N3	1.344 (4)
C2—N1	1.358 (3)	C8—H8	0.9300
C2—C3	1.393 (4)	C9—C10	1.363 (4)
C3—N2	1.367 (4)	C9—H9	0.9300
C3—C4	1.484 (4)	C10—N3	1.346 (4)
C4—O4	1.219 (3)	C10—H10	0.9300
C4—O3	1.306 (4)	C11—N3	1.480 (4)
C5—N2	1.334 (4)	C11—H11A	0.9600
C5—N1	1.358 (4)	C11—H11B	0.9600
C5—C6	1.459 (4)	C11—H11C	0.9600
C6—C9	1.391 (4)	N1—H1	0.8600
C6—C7	1.393 (4)	O3—H3	0.8200
			
O1—C1—O2	125.0 (3)	N3—C8—H8	119.5
O1—C1—C2	117.6 (3)	C7—C8—H8	119.5
O2—C1—C2	117.4 (3)	C10—C9—C6	120.8 (3)
N1—C2—C3	104.7 (3)	C10—C9—H9	119.6
N1—C2—C1	120.5 (2)	C6—C9—H9	119.6
C3—C2—C1	134.8 (3)	N3—C10—C9	120.0 (3)
N2—C3—C2	110.6 (2)	N3—C10—H10	120.0
N2—C3—C4	120.6 (3)	C9—C10—H10	120.0
C2—C3—C4	128.8 (3)	N3—C11—H11A	109.5
O4—C4—O3	121.0 (3)	N3—C11—H11B	109.5
O4—C4—C3	121.5 (3)	H11A—C11—H11B	109.5
O3—C4—C3	117.4 (3)	N3—C11—H11C	109.5
N2—C5—N1	111.1 (3)	H11A—C11—H11C	109.5
N2—C5—C6	123.7 (3)	H11B—C11—H11C	109.5
N1—C5—C6	125.2 (3)	C5—N1—C2	108.5 (2)
C9—C6—C7	117.7 (3)	C5—N1—H1	125.8
C9—C6—C5	119.6 (3)	C2—N1—H1	125.8
C7—C6—C5	122.7 (3)	C5—N2—C3	105.0 (2)
C8—C7—C6	119.6 (3)	C8—N3—C10	120.7 (3)
C8—C7—H7	120.2	C8—N3—C11	119.3 (3)
C6—C7—H7	120.2	C10—N3—C11	119.9 (3)
N3—C8—C7	121.1 (3)	C4—O3—H3	109.5
			
O1—C1—C2—N1	−1.2 (4)	C5—C6—C7—C8	179.8 (3)
O2—C1—C2—N1	177.3 (3)	C6—C7—C8—N3	0.0 (5)
O1—C1—C2—C3	−179.9 (3)	C7—C6—C9—C10	0.0 (4)
O2—C1—C2—C3	−1.5 (5)	C5—C6—C9—C10	−180.0 (3)
N1—C2—C3—N2	0.4 (3)	C6—C9—C10—N3	0.2 (5)
C1—C2—C3—N2	179.2 (3)	N2—C5—N1—C2	−0.4 (3)
N1—C2—C3—C4	178.5 (3)	C6—C5—N1—C2	179.4 (3)
C1—C2—C3—C4	−2.6 (5)	C3—C2—N1—C5	0.0 (3)
N2—C3—C4—O4	1.9 (4)	C1—C2—N1—C5	−179.1 (2)
C2—C3—C4—O4	−176.1 (3)	N1—C5—N2—C3	0.6 (3)
N2—C3—C4—O3	−178.9 (3)	C6—C5—N2—C3	−179.2 (3)
C2—C3—C4—O3	3.1 (5)	C2—C3—N2—C5	−0.6 (3)
N2—C5—C6—C9	2.5 (4)	C4—C3—N2—C5	−178.9 (3)
N1—C5—C6—C9	−177.3 (3)	C7—C8—N3—C10	0.2 (5)
N2—C5—C6—C7	−177.5 (3)	C7—C8—N3—C11	−179.2 (3)
N1—C5—C6—C7	2.7 (5)	C9—C10—N3—C8	−0.3 (4)
C9—C6—C7—C8	−0.1 (4)	C9—C10—N3—C11	179.0 (3)

### Hydrogen-bond geometry (Å, °)

**Table:**

*D*—H···*A*	*D*—H	H···*A*	*D*···*A*	*D*—H···*A*
N1—H1···O1^i^	0.86	1.91	2.764 (3)	174
O3—H3···O2	0.82	1.64	2.461 (3)	176
C7—H7···O1^i^	0.93	2.24	3.145 (4)	165
C8—H8···O3^ii^	0.93	2.58	3.363 (4)	142
C10—H10···O4^iii^	0.93	2.32	3.172 (4)	152
C11—H11C···O4^iii^	0.96	2.35	3.223 (4)	150

Symmetry codes: (i) −*x*, −*y*+1, −*z*+2; (ii) *x*, −*y*+3/2, *z*−1/2; (iii) −*x*+2, −*y*+2, −*z*+2.
